# Diversity and environmental distribution of Asgard archaea in shallow saline sediments

**DOI:** 10.3389/fmicb.2025.1549128

**Published:** 2025-03-18

**Authors:** Karin Hager, Zhen-Hao Luo, Marina Montserrat-Diez, Rafael I. Ponce-Toledo, Pamela Baur, Sven Dahlke, Adrian-Stefan Andrei, Paul-Adrian Bulzu, Rohit Ghai, Tim Urich, Stephan Glatzel, Christa Schleper, Thiago Rodrigues-Oliveira

**Affiliations:** ^1^Department of Functional and Evolutionary Ecology, Archaea Biology and Ecogenomics Unit, University of Vienna, Vienna, Austria; ^2^Department of Geography and Regional Research, Faculty of Earth Sciences, Geography and Astronomy, University of Vienna, Vienna, Austria; ^3^Hiddensee Biological Station, University Greifswald, Greifswald, Germany; ^4^Microbial Evogenomics Lab, Limnological Station, Department of Plant and Microbial Biology, University of Zurich, Zürich, Switzerland; ^5^Department of Aquatic Microbial Ecology, Institute of Hydrobiology, Biology Centre of the Czech Academy of Sciences, České Budějovice, Czechia; ^6^Institute of Microbiology, University Greifswald, Greifswald, Germany

**Keywords:** Asgard archaea, archaeal diversity, microbial ecology, aquatic sediments, 16S rRNA gene

## Abstract

In recent years, our understanding of archaeal diversity has greatly expanded, especially with the discovery of new groups like the Asgard archaea. These archaea show diverse phylogenetic and genomic traits, enabling them to thrive in various environments. Due to their close relationship to eukaryotes, a large number of metagenomic studies have been performed on Asgard archaea. Research on the fine scale distribution, diversity and quantification in saline aquatic sediments where they mostly occur, has, however, remained scarce. In this study, we investigated depths of shallow saline sediment cores from three distinct European environments: the Baltic Sea near Hiddensee, the coastal Lake Techirghiol in Romania, and an estuarine canal in Piran, Slovenia. Based on 16S rDNA amplicon sequencing, we observe variation in the relative abundance and occurrence of at least seven different Asgard groups that are distinct between the three environments and in their depth distribution. Lokiarchaeia and Thorarchaeia emerge as dominant Asgard groups across all sites, reaching maximal relative abundances of 2.28 and 2.68% of the total microbial communities respectively, with a maximal abundance of all Asgard reaching approx. 5.21% in Hiddensee. Quantitative PCR assays provide insights into the absolute abundance of Lokiarchaeia, supporting distinct patterns of distribution across depths in different sediments. Co-occurrence network analysis indicates distinct potential microbial partners across different Asgard groups. Overall, our study shows that Asgard archaea are found as a stable component in shallow sediment layers and have considerably diversified on macro- and microscales.

## Introduction

Archaeal diversity has expanded considerably in the last decades, with numerous novel groups being described (Adam et al., 2017). Culture-independent studies have contributed significantly to the improvement of our understanding of the roles these organisms play in biogeochemical cycles, microbial communities, and evolution (Baker et al., 2020; Tahon et al., 2021). Among the more recently described groups, the Asgard archaea have drawn significant interest due to their close phylogenetic relationship to eukaryotes and their potential role in eukaryogenesis which is further supported by a significant number of Eukaryotic Signature Proteins (ESPs) in Asgard archaeal genomes (Spang et al., 2015; Zaremba-Niedzwiedzka et al., 2017; Lopez-Garcia and Moreira, 2020; Liu et al., 2021; Hatano et al., 2022; Eme et al., 2023).

A growing number of lineages is being discovered within the Asgard archaea and as a consequence, the taxonomic ranks are constantly revised (Rinke et al., 2021; Sun et al., 2021). There are also on-going debates on this group’s taxonomy, where the whole phylum is now being referred to either Asgardarchaeota (Tamarit et al., 2024) or Promethearchaeota (Imachi et al., 2024). The *Lokiarchaeia* (formerly Lokiarchaeota) were the first to be described based on genomic analysis from deep marine sediments (Spang et al., 2015) and evidence suggested the potential for a heterotrophic lifestyle (Spang et al., 2019; Zhang et al., 2021). This was later confirmed by the first lokiarchaeal cultures of *Promethearchaeum syntrophicum* (Imachi et al., 2020; Imachi et al., 2024) and *Ca.* Lokiarchaeum ossiferum (Rodrigues-Oliveira et al., 2023), for which growth on peptides as the major carbon source was observed, consistent with predictions from environmental studies for this lokiarchaeal subgroup (Loki-2b, Yin et al., 2021). However, genome analysis and stable isotope probing of other clades within the *Lokiarchaeia* indicate additional metabolic capabilities, such as those detected for the Loki-3 subgroup (Yin et al., 2021), which includes degradation of lignin and humic acids, CO_2_ assimilation, heterotrophic lactate degradation and aromatic compound degradation, resulting in the possible occupation of distinct ecological niches (Yin et al., 2021). Furthermore, based on genomic data, *Helarchaeales,* which are now considered a lokiarchaeal order, have been described as being capable of hydrocarbon degradation (Seitz et al., 2019), *Thorarchaeia* were suggested to have the potential for mixotrophy (Seitz et al., 2016; Liu et al., 2018), *Wukongarchaeia* for chemolithotrophy (Liu et al., 2021) and *Heimdallarchaeia*, which contains the orders *Gerdarchaeales, Kariarchaeales* and *Hodarchaeales*, have the potential for fermentation as well as aerobic and anaerobic respiration (Spang et al., 2019; Bulzu et al., 2019; Cai et al., 2020; Lu et al., 2021; Liu et al., 2021). Furthermore, *Heimdallarchaeia* have been suggested to participate in nitrogen cycling, as both nitrate and nitrite reductases have been detected in genomes from organisms of this group (MacLeod et al., 2019), while select thorarchaeal genomes harbor nitrogenases, hinting at a role in assimilating atmospheric nitrogen into ammonia (Wong et al., 2018; Liu et al., 2018). Asgard archaea have also been suggested to participate in assimilatory sulfate reduction and activation, due to the detection of sulfate adenylyltransferases and phosphoadenosine phosphosulfate reductases in their genomes (Zaremba-Niedzwiedzka et al., 2017; MacLeod et al., 2019).

The reconstruction of metabolic diversity described within the Asgard archaea is consistent with their worldwide distribution, as a large number of metagenome-assembled genomes (MAGs) have been retrieved not only from a variety of anoxic sediments with consistently higher abundances in saline environments (Zaremba-Niedzwiedzka et al., 2017; Seitz et al., 2019; Bulzu et al., 2019; Zou et al., 2020), but also from other natural ecosystems such as soils, plant rhizospheres, and hot springs (Zaremba-Niedzwiedzka et al., 2017; Cai et al., 2021). These studies show that *Lokiarchaeia* and *Thorarchaeia* are more widely distributed, while *Heimdall-* and *Odinarchaeia* were found to be more restricted, with the former typically occurring in more saline environments and the latter in thermophilic ones (Cai et al., 2021), which is in line with our previous studies showing *Lokiarchaeia* in environments with a wider pH range in comparison to other Asgard groups (Manoharan et al., 2019). Considering the distribution and abundance of Asgard archaea in selected sediments (Inagaki et al., 2003; Jørgensen et al., 2013; Saghaï et al., 2017; MacLeod et al., 2019) that can even represent the most abundant microorganisms in some deeper layers with >50% of all prokaryotic 16S rRNA genes (“DSAG” group in Jorgensen et al., 2012), it is likely that these organisms display an important role in carbon cycling at least in such environments.

Considering their importance in both evolutionary and environmental contexts, and in light of the fact that only two organisms of the whole phylum have been cultivated so far (Imachi et al., 2020; Rodrigues-Oliveira et al., 2023), exploring Asgard archaeal diversity in easily accessible environments with relatively high abundance is imperative. In this study shallow marine sediments from three different European environments were investigated: the Baltic Sea in the proximity of the German island Hiddensee, the Romanian Salt Lake Techirghiol (coastal barrier lake formed by the Black Sea), and an estuarine canal in Piran, Slovenia, from where *Ca.* L. ossiferum was first enriched (Rodrigues-Oliveira et al., 2023). Our 16S rRNA gene analyses reveal that up to seven different Asgard classes inhabit these sediments. We explore the distinct populations in these habitats and search for the occurrence of a few cosmopolitan lineages.

## Materials and methods

### Sediment core sampling sites and processing

Sediment cores were sampled from three different environments in duplicates. Marine sediments from the Baltic Sea were sampled in October 2019, in the lagoon (“bodden”) approximately 300 m from the shore of the German island Hiddensee (54°34′51.2”N 13°07′50.6″E). The water depth was about 3 meters and the water temperature was 15°C. The second sampling site was Lake Techirghiol, the largest salt water lake in Romania located in a coastal area around 150 m west of the Black Sea. Sediment cores were retrieved on March 2020 about 20 meters away from the shore (44°03′11.5”N 28°36′12.7″E), where the water depth was approximately 1 meter and its temperature was 12°C. The third site was an estuarine canal in Piran, Slovenia (45°29′45.78”N 13°36′10.08″E), where sediment cores were sampled 3 meters from the shore in May 2022. The water depth was about 0.5 meters and the temperature was 27°C. On all sites, the cores were between 25 and 30 cm in length. They were processed inside an anaerobic chamber (N_2_ atmosphere), where they were cut at 2 cm intervals. Each fraction was then placed in 50 mL conical sterile centrifuge tubes without the addition of any solutions, sealed with parafilm and stored at 4°C until further analyses, which were performed shortly after to avoid sample degradation.

### Sediment analyses of physico-chemical parameters

The physico-chemical analyses were performed at specific depth-sections of the sediments, depending on the amount of sample required for each analysis and the sampling depth of the cores, but mainly in the following depth-sections per sediment core: 0–2 cm, 4–6 cm, 8–10 cm, 20–22 cm and 24–26 cm. For the Hiddensee site, the second core samples intended for physical–chemical analyses were unfortunately lost during transportation.

The gravimetric water content (θ_g_) was determined by completely drying the weighed wet sample (m_w_) in the freeze dryer to constant dry mass (m_d_) and was calculated as follows:


$$\theta_{g}=\frac{m_{w}-m_{d}}{m_{w}}*100\%$$

The completely dried samples were homogenized with a 2 mm sieve and pulverized with a mixer mill (MM400, RETSCH GmbH, Haan, Germany). The LECO RC612 multiphase carbon analyzer (LECO Europe B.V., AG Geleen, Netherlands) was used to determine the total organic (TOC, ≤ 450°C), the total inorganic (TIC, > 450°C & ≤ 1,000°C) and the total carbon content (TC, sum of TOC and TIC) of the sediment samples (in percent per dry weight) via temperature-dependent CO_2_ measurement (dry combustion method).

For the determination of pH and electrical conductivity (EC), the samples were air-dried at 40°C until mass stability and subsequently sieved (< 2 mm). The sieved samples were then incubated overnight with ultrapure water (ratio 1:10 w/v), shaken for 1 h in an overhead shaker, and left standing for 30 min prior to measurements of pH (pHmeters pH 7,110 and pH 720, WTW GmbH, Weilheim, Germany) and EC (Cond 7,110, Xylem Analytics Germany GmbH, Weilheim, Germany).

The grain size of the fine sediment samples (<2 mm) was determined by sieving and pipetting (sedimentation): the sand fractions (<2 mm & ≥ 0.063 mm) by sieving and the silt fractions (< 0.063 mm & ≥ 0.002 mm) as well as clay content (< 0.002 mm) by pipetting, both according to ÖNORM L 1061-2 (Austrian Standards International, 2019). Due to the high salt content of the samples, washing was carried out before the analyses. For this purpose, ultrapure water was added to each sample, mixed, and centrifuged at 5000 rpm for a maximum of 30 min. The resulting clear liquid was poured off and the washing cycle was repeated until the EC value of the samples had dropped to <100 μS cm^−1^. As additional preparation, humus destruction with hydrogen peroxide (15%) was carried out on each sample. The resulting proportions of the respective grain size are reported as a percentage of the air-dried fine sediment.

### 16S rRNA gene amplification, sequencing and analysis

DNA extraction protocol used 0.5 g of each sediment fraction using the DNeasy PowerSoil Pro Kit (Qiagen) according to the manufacturer’s instructions. DNA concentration was measured in a Qubit 2.0 Fluorometer (Invitrogen), using the dsDNA HS kit. Prokaryotic 16S rRNA genes were amplified by PCR using the Earth Microbiome Project general prokaryotic 16S rRNA gene targeting primer pair 515f (5′- GTG CCA GCM GCC GCG GTA A) and 806r (5′- GGA CTA CHV GGG TWT CTA AT) as previously described (Caporaso et al., 2011), which were then barcoded and subjected to paired-end sequencing at the Vienna BioCenter Core Facilities (VBCF) using the Illumina Miseq (300 PE) platform, resulting in 13.2–15 Gb of raw data per flowcell. The Illumina adaptor and primer sequences were trimmed from raw fastq sequences with Cutadapt v. 4.7 (Martin, 2011). Subsequently, sequences were processed following the DADA2 pipeline (Callahan et al., 2016) in R v. 4.2.1 in order to obtain quality trimmed nonchimeric sequences and generate amplicon sequencing variants (ASVs). The taxonomic classification was assigned with the RDP method in DADA2, using the GTDB (Parks et al., 2022) 16S rRNA gene DADA2 adapted database v.4.2 (Ali, 2023). The occurrence of the specific ASV was evaluated by screening with the IMNGS platform (Lagkouvardos et al., 2016). The 16S rRNA gene sequencing data generated and analyzed in this study are available in the NCBI database under BioProject accession number PRJNA1068488.

### Quantitative PCR

Lokiarchaeal 16S rRNA gene quantitative PCR (qPCR) assays were performed using primers LkF (5’ ATC GAT AGG GGC CGT GAG AG) and LkR (5’ CCC GAC CAC TTG AAG AGC TG) as previously described (Rodrigues-Oliveira et al., 2023). These primers have a 79.9% coverage of the *Lokiarchaeia* taxonomy according to the SILVA TestPrime tool when allowing 1 mismatch (Klindworth et al., 2012). Lokiarchaeal 16S rRNA genes were amplified from environmental DNA and cloned using the CloneJET PCR Cloning Kit (Thermo Scientific). Following transformation into *E. coli*, successful cloning of the target gene was confirmed via Sanger sequencing. Plasmids were then extracted and quantified using the Qubit 2.0 Fluorometer (Invitrogen). The quantified plasmids were subsequently serially diluted from 10^1^ to 10^8^ copies/mL to serve as quantification standards. The efficiencies of these reactions varied from 90 to 100%, with *R*^2^ values >0.99.

### Phylogenetic tree of 16S rRNA gene sequences

Asgard ASVs were aligned to a reference Asgard 16S rRNA alignment (Liu et al., 2021) using MAFFT 7.520 (Katoh and Standley, 2013) (--addfragments option, L-INS-i algorithm). IQ-TREE v2.2.2.7 (Minh et al., 2020) was used to reconstruct the maximum likelihood phylogenetic tree with the best-fit model SYM + R5 chosen by ModelFinder (“-m MFP”) (Kalyaanamoorthy et al., 2017) according to BIC (Bayesian Information criterion), 1,000 ultrafast bootstrap replicates (“-bb 1,000”) and SH-like approximate likelihood ratio test (“-alrt 1,000”) (Guindon et al., 2010). The tree was visualized with the Interactive Tree Of Life (iTOL) software (Letunic and Bork, 2021). Except for *Lokiarchaeia* with subgroup differentiation, genus level taxonomic assignment of the other Asgard ASVs was determined by the combination of the DADA2 pipeline and their phylogenetic relatedness to Asgard lineages.

### Diversity and statistical analysis

To allow comparison on an equal basis, data were rarefied to the lowest reads (32,297) per sample (*n* = 26) for the downstream comparison analyses. Downstream analysis and visualization were performed by different packages in R v. 4.2.1. Data management and visualization of ASV relative abundances was done with phyloseq (McMurdie and Holmes, 2013) and ggplot2 (Wickham, 2011). To assess alpha diversity (within sample diversity) we calculated Shannon and Inverse Simpson indexes with the ampvis2 package and Faith’s diversity with the picante package. To compare alpha diversity metrics between groups we performed a Wilcoxon rank sum test, adjusting the *p*-value with the Benjamini-Hochber (B-H) method. For the beta-diversity analysis, (between sample diversity), a distance matrix using Bray-Curtis distances was calculated by obtaining the average values of 100 iterations of random subsamples (*n* = minimum sequencing depth) of the ASVs counts matrix distance, with the vegan function avgdist. Beta diversity was visualized in a non-metric multidimensional scaling (NMDS) with the metaMDS function and evaluated with permutational multivariate ANOVA (PERMANOVA) (Tang et al., 2016) with 999 permutations, performed with the adonis function of the vegan R package. Pearson correlations were conducted using the stats package to assess the relationships between Asgard Archaea composition and physico-chemical parameters with cor.test function. Furthermore, to explore the potential environmental parameters driving the microbial community structure Canonical Correspondence Analysis (CCA) models were built. For that we used an environmental data matrix with soil physicochemical parameters and the ASV counts matrix. In order to take into account the multicollinearity between the environmental parameters, we used a stepwise selection approach based on the variance inflation factor, calculated by the vif.cca function in the vegan package, selecting the variables with a value under 20. The model was tested for statistical significance with an ANOVA-like permutation test, implemented with the anova.cca function in the vegan package.

### Microbial co-occurrence network analysis

The potential interaction relationships between the Asgard archaea and other microorganisms was investigated by applying SparCC (Sparse Correlations for Composition data) network (Friedman and Alm, 2012) analysis to ASVs levels. Only ASVs with a minimum of 10 reads and detected in at least 20% of the samples were used for network construction to prevent spurious associations caused by ASVs with low abundances. The network was generated using 1,000 bootstraps and filtered by *p* value <0.05. The positive relationships (correlation >0) between Asgard archaea and ASVs were summarized at the class level. Specially, the subgroups (Loki1, Loki2a, Loki2b, Loki3) of Lokiarchaeia were classified with phylogenetic relatedness to a reference Lokiarchaeia 16S rRNA database via phylogenetic tree analysis as described above (Yin et al., 2021). Only the groups with highest number of potential interactions (Top 10) with Asgard archaea was shown and network visualization was conducted in Cytoscape (Shannon et al., 2003).

## Results

### Sediment characterizations

**Figure 1:**
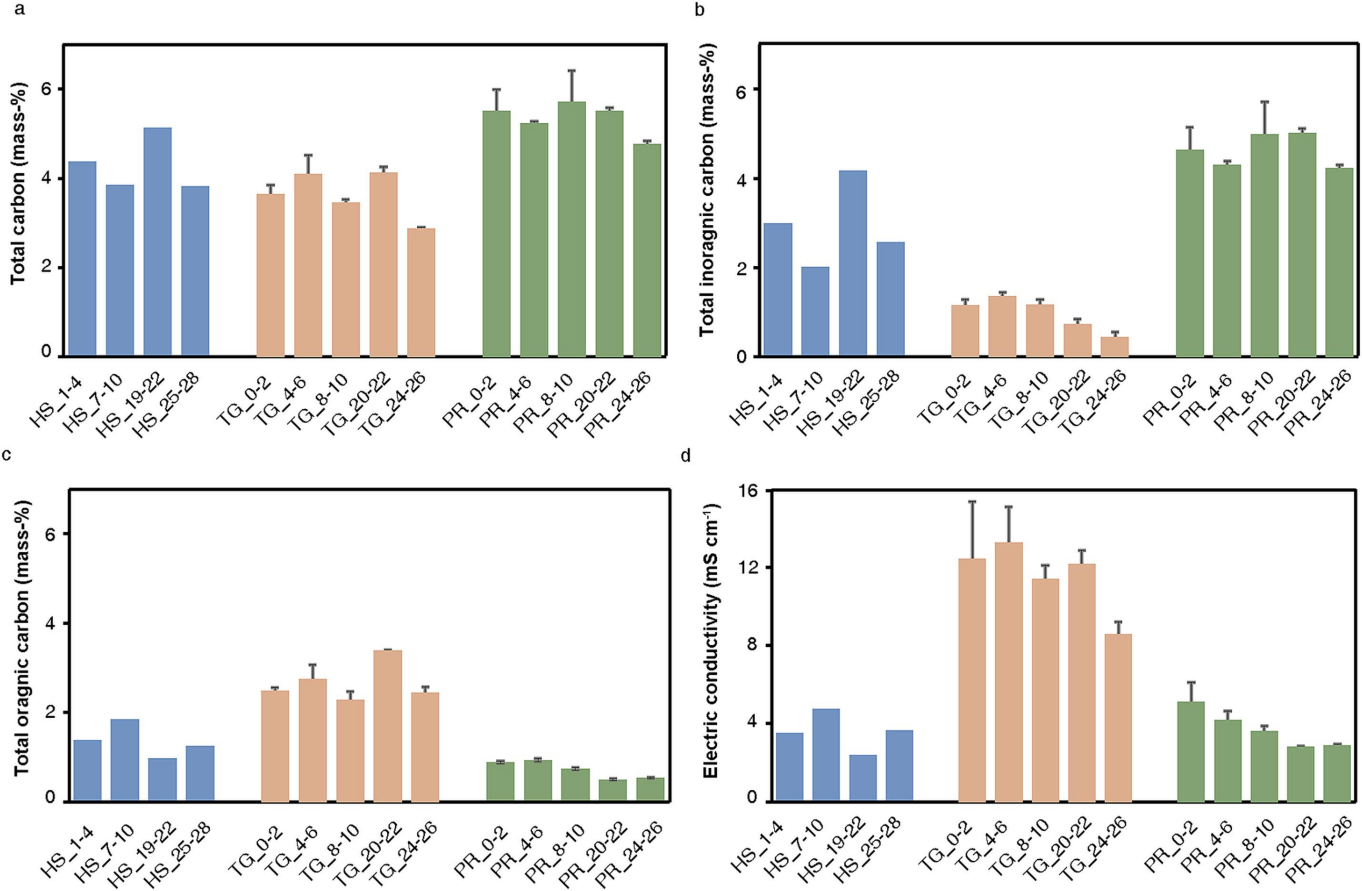
Physico-chemical parameters measured at Hiddensee, Piran and Lake Techirghiol. We measured Total Carbon **(a)**, Total Inorganic Carbon **(b)**, Total Organic Carbon **(c)** and Electric conductivity **(d)**. Different colors represent different sites (blue: Hiddensee, orange: Techirghiol, green: Piran).

Sediment cores (25–30 cm) were taken in duplicates from three different marine locations across Europe, including a shallow lagoon of Hiddensee island in the Baltic Sea, Germany, the coastal lake Techirghiol in Romania, and a small estuarine canal that receives regular inflow from the Mediterranean Sea in Piran, Slovenia. Various parameters were measured across sediment layers in this study to establish an environmental context for the identified Asgard archaea communities. These parameters included pH, total carbon (TC), total organic carbon (TOC), total inorganic carbon (TIC), gravimetric water content (θ_g_), grain size and electric conductivity (EC), the latter being interpreted as a proxy for salinity (Figure 1).

Our analyses indicate that the pH levels across all three sites were similar, hovering around 8.6 ± 0.3 (mean ± SD) at all depths. While there was no stratification in total carbon content at the sampling sites (Figure 1a), variations in total inorganic (Figure 1b) and organic (Figure 1c) content and were evident among sediments from different sites. Samples from Hiddensee and particularly Piran exhibited higher TIC levels (4.6 ± 0.5 mass-%), than Lake Techirghiol, which displayed higher TOC (2.7 ± 0.4 mass-%). TIC content in Lake Techirghiol decreased with depth. Additionally, this sampling site showed the highest electrical conductivity (EC) (Figure 1d) among the three sites, reaching values of up to 14.6 mS cm^−1^, while sediments from Piran and Hiddensee contained lower concentrations of dissolved salts in comparison. In contrast to the other sites, the sediments of Lake Techirghiol were characterized by finer grain sizes such as silt and clay rather than sand, regardless of depth (Supplementary Figure 1). Therefore, this site showed also the highest gravimetric water content θ_g_ (43.8 ± 5.3 mass-%).

### Microbial and Asgard archaea diversity analyses

The 25 to 30 cm long cores were cut into layers of 2 cm of which DNA was prepared for 16S rDNA amplicon sequencing using the primers of the Earth microbiome project for *Bacteria* and *Archaea* (Caporaso et al., 2011). After processing, around 112,028 reads were recovered per sample, (mean value; 2,912,721 in total) and used for further analyses.

**Figure 2:**
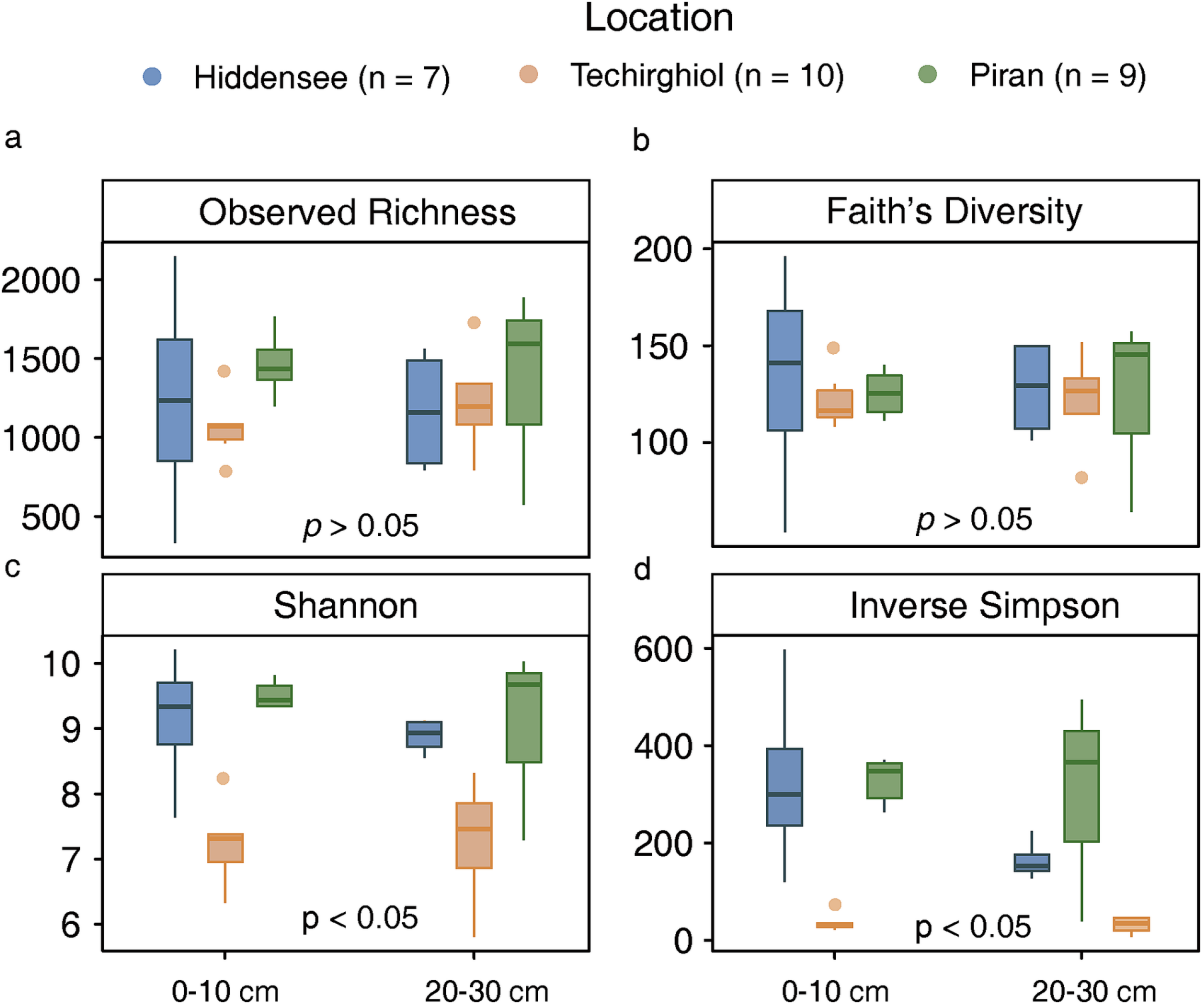
Comparisons between observed richness **(a)**, Faith’s diversity **(b)**, Shannon index **(c)** and inverse Simpson index **(d)** of 16S rRNA gene sequences from the sediment microbiome in Hiddensee, Piran, and Lake Techirghiol. Different colors represent different samples. (blue: Hiddensee, Orange: Techirghiol, Green: Piran).

The alpha diversity indices including observed richness (Figure 2a), Faith’s diversity (Figure 2b), Shannon index (Figure 2c), and inverse Simpson index (Figure 2d) showed different distribution patterns among different locations. As indicated by the inverse Simpson and Shannon indices, which inform of both the richness and evenness of species in the microbial communities, the microbial communities in Piran and Techirghiol harbor more diverse and even assemblages than those in Hiddensee (Wilcoxon test, *p*-value <0.05). However, this trend does not appear in observed richness and Faith’s diversity indexes, indicating that there were no significant differences between the sampling sites when considering the phylogenetic diversity pool and binary presence-absence data. Besides, the Hiddensee microbial community was richer in superficial layers (< 10 cm) than in the deeper ones (>20 cm) based on the inverse Simpson index, though not statistically supported.

**Figure 3:**
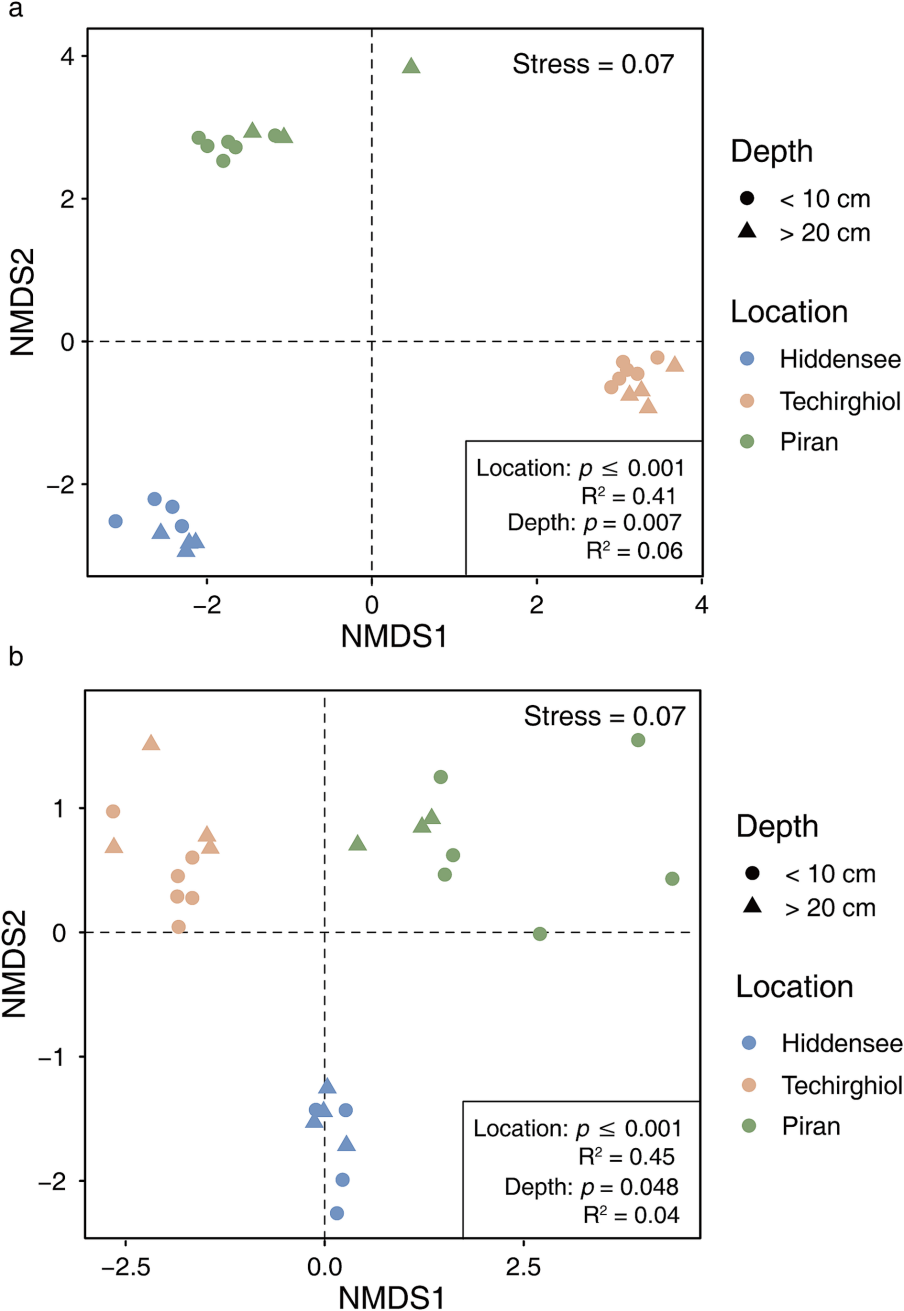
Non-metric multidimensional scaling (NMDS) based on Bray–Curtis dissimilarity matrix for the whole microbial community **(a)** and Asgard archaeal **(b)** 16S rRNA genes. The analysis of similarity (ADONIS) statistics considers samples from different locations and depths.

NMDS analysis revealed that the microbial community structure was clearly separated between the three sediment locations (PERMANOVA: *R*^2^ = 0.41, *p* < 0.05, Figure 3a). Notably, this significant distinction extended to the Asgard archaea communities within these environments (PERMANOVA: *R*^2^ = 0.45, *p* < 0.05, Figure 3b). Besides, the disparities were also observed between different depths within the general (PERMANOVA: *R*^2^ = 0.06, *p* = 0.007, Figure 3a) and Asgard archaea community level (PERMANOVA: *R*^2^ = 0.04, *p* = 0.075, Figure 3b), though with lower statistical significance.

### Asgard archaea diversity and abundance in sediment layers

**Figure 4:**
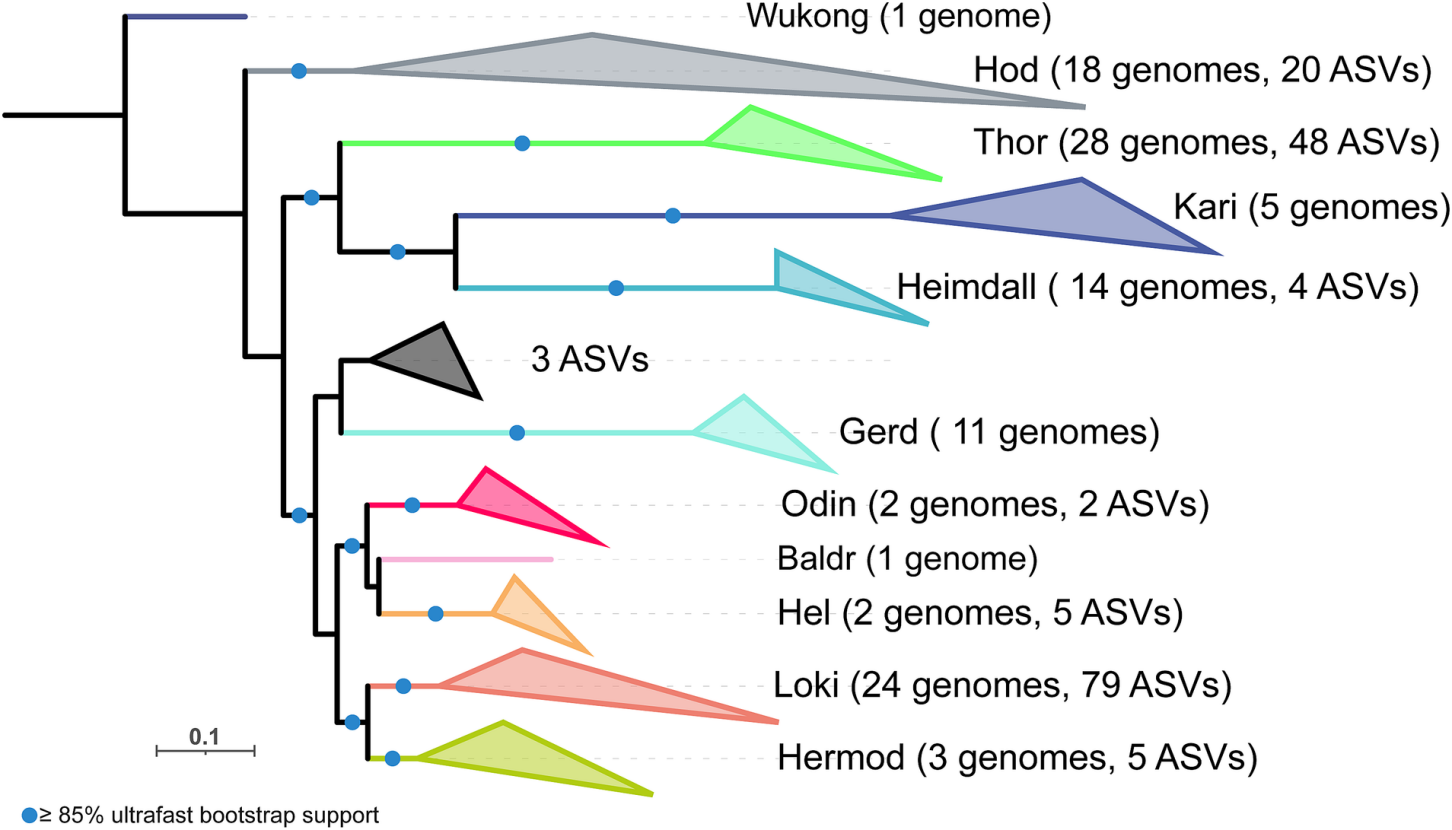
Phylogenetic tree of Asgard archaea 16S rRNA genes (Liu et al., 2021) and ASVs obtained from the Hiddensee, Lake Techirghiol and Piran (see Supplementary Figure 2). The scale bar indicates the number of substitutions per site. Baldr, *Baldrarchaeia*; Gerd, *Gerdarchaeales*; Hel, *Helarchaeales*; Heimdall, *Heimdallarchaeia*; Hermod, *Hermodarchaeia*; Hod, *Hodarchaeales*; Kari, *Kariarchaeaceae*; Loki, *Lokiarchaeia*; Odin, *Odinarchaeia*; Thor, *Thorarchaeia*; Wukong, *Wukongarchaeia*.

**Figure 5:**
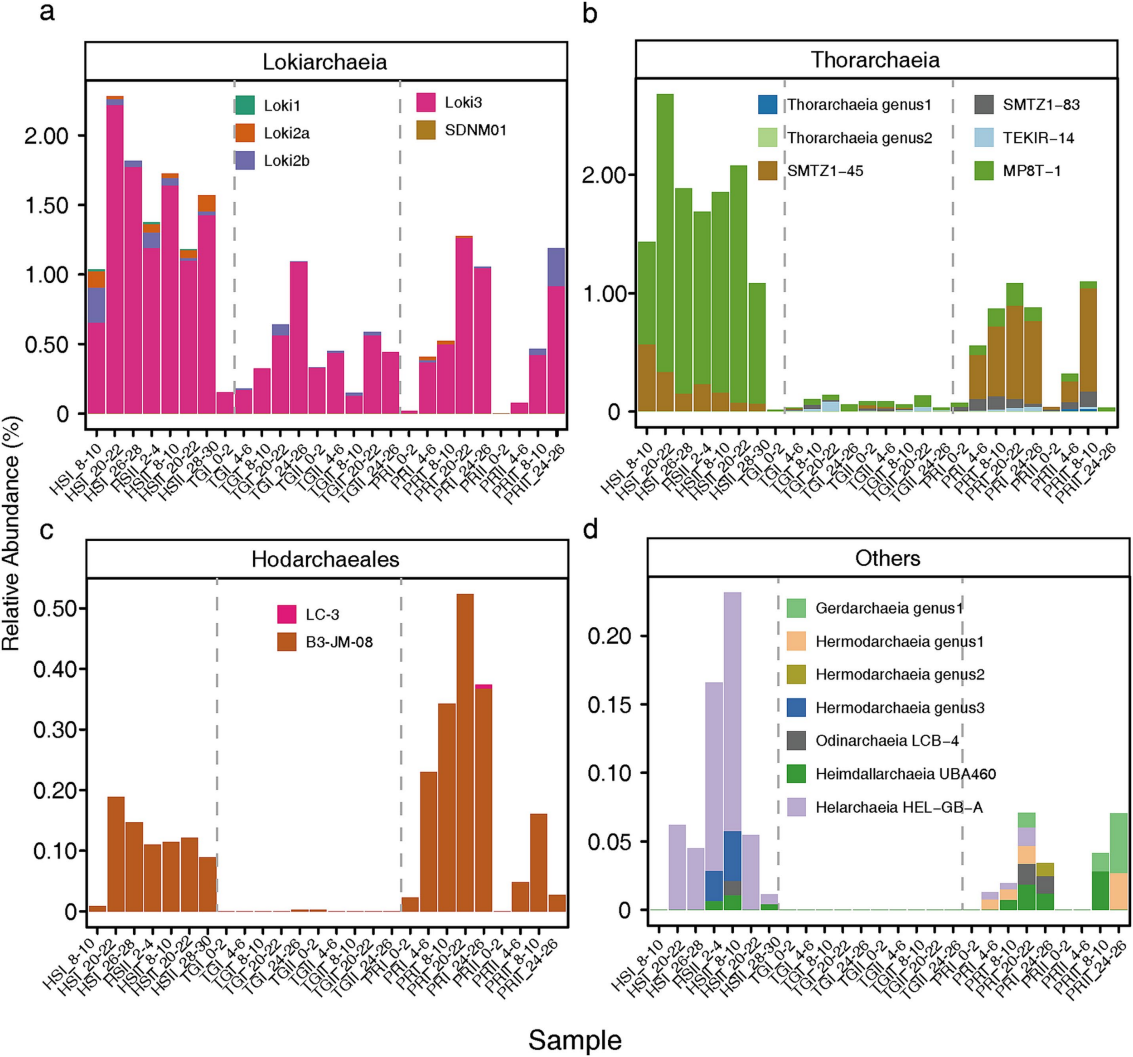
Asgard archaea microbial diversity profile in cores from Hiddensee (HSI and HSII), Piran (PRI and PRII), and Lake Techirghiol (TGI and TGII) at different depths. Different colors represented different Asgard groups: Lokiarchaeia **(a)**, Thorarchaeia **(b)**, Hodarchaeales **(c)** and others **(d)**.

All 37,349 reads that were assigned to Asgard archaea were retrieved from the dataset and subjected to clustering into Amplicon Sequence Variants (ASVs). Subsequently, taxonomic ranks were assigned based on the combination of their taxonomic information generated by DADA2 and their placement in an updated reference 16S rRNA gene phylogenetic reconstruction (Liu et al., 2021) (Figure 4; Supplementary Figure 2). The distribution of Asgard archaea in the three environments varied by location and depth, as illustrated in Figure 5. *Lokiarchaeia* were consistently abundant across all samples (Figure 5a), with the mixotrophic Loki3 being the dominant subgroup, followed by the peptide degrading Loki-2b (Yin et al., 2021), which includes the two currently cultured lokiarchaea (Imachi et al., 2020; Rodrigues-Oliveira et al., 2023). Interestingly, though abundant in Hiddensee and Piran, the dominant genus of *Thorarchaeia* was different in these two sampling sites, with MP8T-1 in Hiddensee and SMTZ1-45 in Piran (Figure 5b). Despite this variation, both groups were originally sampled in estuarine environments and are believed to share highly similar metabolic capabilities, possessing genes associated with peptide degradation and mixotrophy (Seitz et al., 2016; Liu et al., 2018), leaving open the question of what drives their distinct presence in each location.

**Figure 6:**
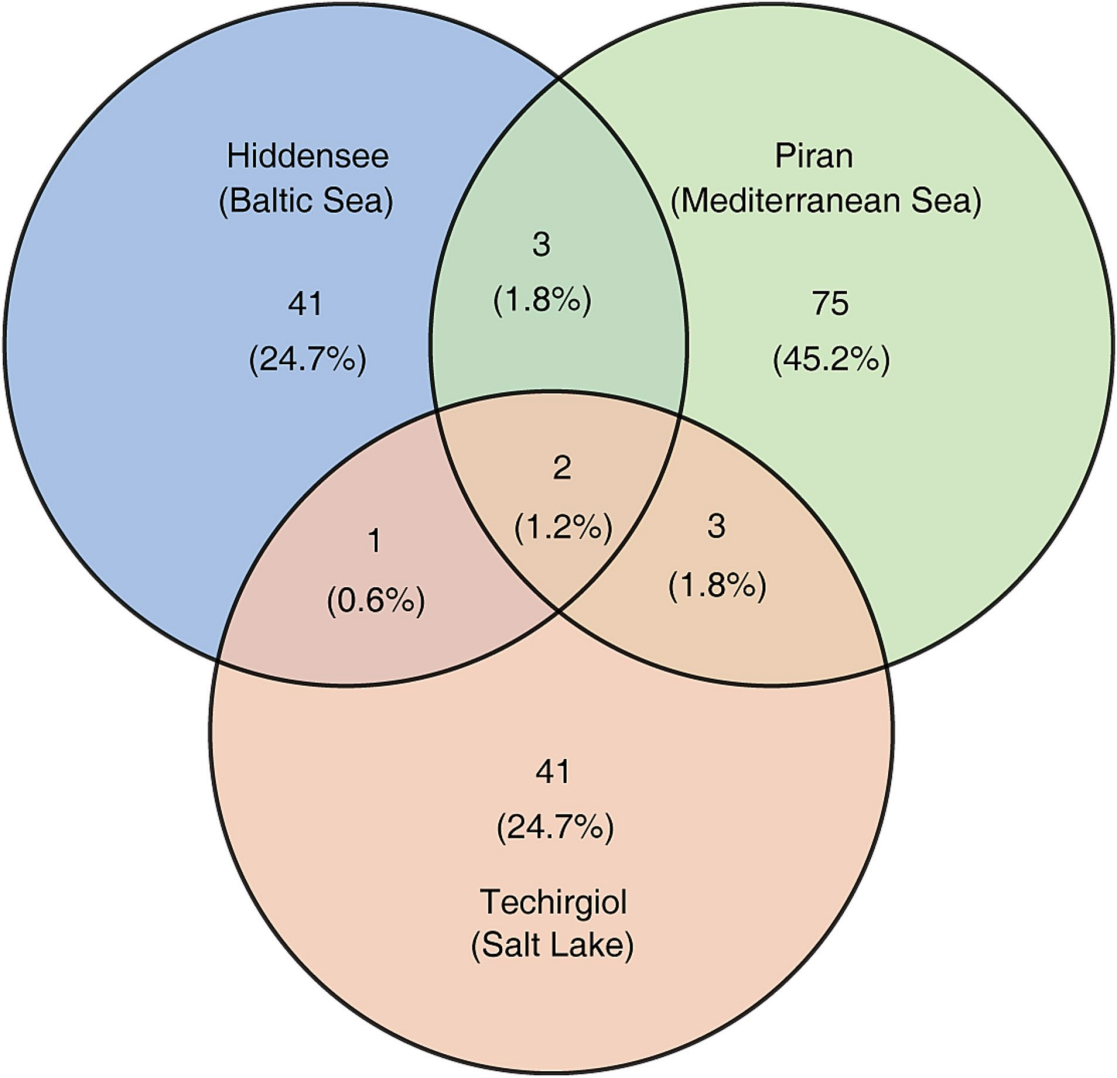
The unique and shared Asgard archaeal 16S rRNA ASVs across samples obtained from the Hiddensee, Piran, and Lake Techirghiol. The numbers in parenthesis indicate the proportions of all Asgard archaeal ASVs detected in this study.

Sediments from Hiddensee exhibited the highest relative abundance of Asgard archaea in the total prokaryotic community, ranging from 1.97 to 5.21%. Alongside *Lokiarchaeia* and *Thorarchaeia*, *Hodarchaeales* were also detected, where the B3-JM-08 genus occupied most of the population in all different samples (Figure 5c). At lower abundance, other groups (Figure 5d) like *Helarchaeia* reached a maximum value of 0.17% in Hiddensee. *Hermodarchaeia* had its highest detection in a Hiddensee core at depths ranging between 7 and 10 cm, dominated by one genus. Piran samples displayed the second-highest relative abundance of Asgard archaea (0.04 to 2.95%). Interestingly, SMTZ1-45 surpassed Loki3 in the upper layers, with Loki3 increasing in abundance with depth. B3-JM-08, the major group of *Hodarchaeales* in our study initially less abundant in the upper layers, exhibited increased values starting from 4–6 cm depths, with Piran having the highest relative abundance of this group (up to 0.52% in selected layers). Compared to Hiddensee, *Helarchaeia* HEL-GB-A were detected in fewer samples and at lower amounts (two samples as opposed to six, with a maximum relative abundance of 0.14%). Lastly, Lake Techirghiol displayed the lowest relative abundance of Asgard archaea (0.17 to 1.15%), with higher values predominantly detected in the lower layers. It was also identified as the least diverse environment, primarily featuring Loki3 and *Thorarchaeia* across all depths, with Loki3 being more abundant overall. Furthermore, it is noteworthy that only a very small number of ASVs were common across all sampling sites, with the vast majority being unique to each individual location (Figure 6). Both ASVs that were shared across all three sites belong to the Loki-3 subgroup. Most of the ASVs shared between the site pairs—Hiddensee and Piran, Hiddensee and Techirgiol, and Piran and Techirgiol—also belong to this group, with two exceptions: the Hiddensee-Piran pair includes one shared ASV from the *Thorarchaeia*, and the Techirgiol-Piran pair includes one shared ASV from the Loki-2 subgroup (Supplementary Table 1). Albeit with common detections along the different depths, the ASVs shared by the three different sampling sites showed distinct distribution patterns when compared to sequences deposited in databases, where ASV47 was detected in more diverse samples than ASV306 (Supplementary Table 2).

To investigate the potential influence of the measured physicochemical parameters on the Asgard archaeal community, we conducted Pearson correlation analysis (Supplementary Table 3) and Canonical Correspondence Analysis (CCA) (Supplementary Figure 3; Supplementary Table 4). In Pearson correlation analysis, statistically significant relationships (*p* < 0.05) revealed a positive association between *Heimdallarchaeia* and *Hodarchaeales* with TIC and TC, whereas *Lokiarchaeia* and *Thorarchaeia* exhibited a negative correlation with EC, with *Thorarchaeia* also showing a negative relationship with TOC. In the CCA, TIC, TOC, and water content were identified as the strongest determinants for the overall microbial community (Supplementary Figure 3a), while TC, TOC, and EC were the primary factors influencing the Asgard archaeal community (Supplementary Figure 3b), as indicated by the longest arrows in the respective analyses. Overall, water content, EC, pH, and TOC were positively correlated with samples from Techirghiol and negatively correlated with those from Piran and Hiddensee, whereas TC exhibited the opposite pattern.

**Figure 7:**
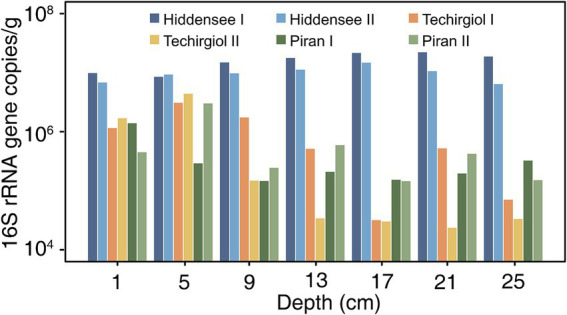
qPCR of lokiarchaeal 16S rRNA gene copies per gram of sediment samples from different environments. Different colors represented different cores.

To obtain absolute quantification estimates for the most widespread lineage of Asgard archaea (*Lokiarchaeia*), we performed qPCR assays using *Lokiarchaeia*-specific 16S rRNA gene primers (Figure 7). Notably, Hiddensee samples exhibited consistent amounts across all depths, ranging from 7.0 × 10^6^ to 2.0 × 10^7^ lokiarchaeal 16S rRNA gene copies per gram of sediment. In contrast, Piran samples displayed higher numbers in the upper layers (approximately 1.0 to 5.0 × 10^6^ copies/g), with a noticeable decrease with depth (approximately 2.0 × 10^4^ to 5.0 × 10^5^ copies/g). Lake Techirghiol, while also showing higher numbers in the upper layers (around 3.0 × 10^5^ to 3.0 × 10^6^ copies/g), exhibited a less pronounced decline with increasing depth when compared to Piran (approximately 1.0 × 10^5^ to 5.0 × 10^5^ copies/g). Akin to the trends observed in relative abundance estimations, the Hiddensee samples consistently exhibited the highest overall values in this quantitative approach.

### Co-occurrence patterns

**Figure 8:**
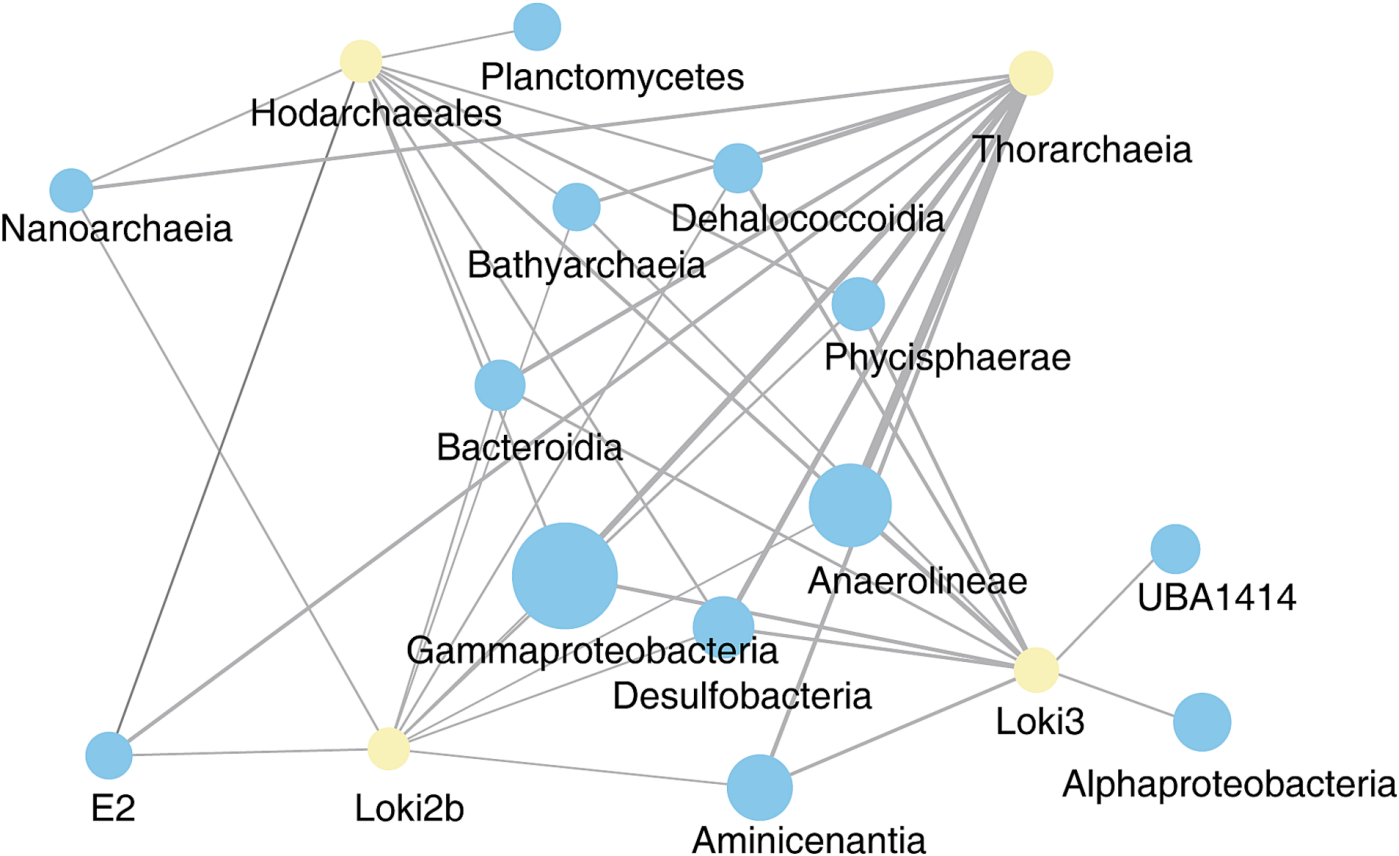
Co-occurrence network (*p* < 0.05) of Asgard archaea (yellow). For visualization, only the microbes (blue) with top 10 number of links to Asgard archaea were included. The size of each node is proportional to the relative abundance of the group and the thickness of each edge is proportional to the number of links between the nodes.

To investigate the significant non-random relationships between Asgard archaea and other lineages, a network interface of microbial communities in the sediment cores was constructed aiming to unveil possible ecological interactions in nature (Cai et al., 2021). At first, we have obtained 1,398 nodes (ASVs) and 13,2,305 edges. After clustering and filtering, a co-occurrence network with 101 nodes (classes) and 290 edges was generated, where only 4 Asgard groups were included (*Lokiarchaeia* 2b, *Lokiarchaeia* 3, *Thorarchaeia,* and *Hodarchaeales*). We could not obtain the co-occurrence patterns of other Asgard groups due to their limited sample distribution in this study. In the network, the groups with the highest number of relationships with Asgard archaea included *Gammaproteobacteria*, *Anaerolineae*, *Phycisphaerae*, *Bathyarchaeia*, *Dehalococcoidia*, *Desulfobacteria*, *Bacteroidia*, *Planctomycetes*, *Nanoarchaeia*, E2, *Aminicenantia*, *Alphaproteobacteria*, and UBA1414, with the former 7 groups shared by all 4 Asgard archaea (Figure 8; Supplementary Table 5). The number of relationships involved in *Thorarchaeia* (mean: 16.8) is higher than other Asgard archaea (mean: 7.2), indicating that this group might be highly dependent on other community members, partially explaining the difficulty in obtaining enrichments or isolates.

## Discussion

In this study, we assessed the diversity and distribution of Asgard archaea across three shallow saline environments in Europe. Each environment served as a distinct representation, differing notably in factors such as salinity and carbon content (Figure 1). The samples from the Hiddensee comprised marine sediments, those from Piran represented an estuarine canal, and Lake Techirghiol exemplified a Salt Lake. These environmental distinctions were clearly manifested in the microbial communities, as indicated by diversity analyses (Figures 2, 3), revealing a distinct separation between samples based on their respective sampling sites. This variation was evident both at the overall community level and specifically within the Asgard archaea population (Figure 5).

The majority of Metagenome-Assembled Genomes (MAGs) and 16S rRNA gene sequences of Asgard archaea have been predominantly derived from saline sediments (Spang et al., 2015; Seitz et al., 2016; Manoharan et al., 2019; Ramírez et al., 2020; Cai et al., 2020). It has also been established that this parameter plays a key role in shaping Asgard archaea diversity within specific environments (Cai et al., 2021). Moreover, as previously noted from genomic studies, the Asgard group includes high metabolic diversity (Spang et al., 2019; Liu et al., 2021), encompassing also different strategies of carbon acquisition. In our samples, *Lokiarchaeia* and *Thorarchaeia* consistently emerge as the dominant groups in all environments (Figures 5a,b), which has been established in other studies (Manoharan et al., 2019; Cai et al., 2021). It is worth noting that while the Loki-3 subgroup dominated all samples, the Loki-2 subgroup, which contains both cultivated lokiarchaeal strains (Imachi et al., 2020; Rodrigues-Oliveira et al., 2023), appeared in higher abundances only in specific layers of cores from Hiddensee and Piran, particularly in the 8–10 cm layer of Hiddensee Core I and the 24–26 cm layer of Piran Core II. Although the widespread presence of Loki-3 aligns with its versatile metabolism and broad ecological distribution, as described by Yin et al. (2021), variations within this subgroup may exist, as suggested by the broader distribution of ASV47 compared to ASV306 (Supplementary Table 2). CCA analysis (Supplementary Figure 3) indicated that TOC was positively correlated with the Asgard archaea community in Techirghiol but negatively correlated in other environments where TC was the major determinant. Given that the Loki-3 subgroup constitutes the majority of the *Lokiarchaeia* community and is capable of both heterotrophic growth via lactate degradation and CO₂ assimilation (Yin et al., 2021), its metabolic flexibility may explain its presence across all sampled environments despite their distinct physicochemical conditions.

Additionally, the dominant *Thorarchaeia* genera differed between the two sites, with MP8T-1 being more abundant in Hiddensee and SMTZ1-45 prevailing in Piran. As previously mentioned, both groups were originally described from estuarine environments and are thought to be mixotrophs (Seitz et al., 2016; Liu et al., 2018). Given that Pearson correlation analysis indicated a negative correlation between *Thorarchaeia* and TOC in our samples (Supplementary Table 3), along with their hypothesized mixotrophy, it is possible that these organisms relied on inorganic carbon metabolism for growth, as ribulose bisphosphate carboxylase-like proteins (without RuBisCO activity) and a nearly complete Calvin–Benson–Bassham cycle were identified in thorarchaeal genomes (Liu et al., 2018).

Piran displayed the highest relative abundance of *Hodarchaeales* when compared to the other two environments (Figure 4c). This finding is interesting, given that this group is often associated with facultative aerobic heterotrophic metabolism (Liu et al., 2021; Eme et al., 2023) and Piran does not boast the highest TOC. Furthermore, Pearson correlation analyses (Supplementary Table 3) did not correlate this factor with this group and CCA plots (Supplementary Figure 3; Supplementary Table 4) did not identify this factor as influencing the Asgard diversity in Piran. Moreover, *Heimdallarcheia*, a related lineage, also had its highest relative abundance detected in Piran and has been suggested to be positively correlated with higher salinity (MacLeod et al., 2019; Cai et al., 2021). While Lake Techirghiol had both higher TOC and EC values, these specific Asgard archaeal groups were not detected there. Pearson correlation analyses (Supplementary Table 3) revealed a positive correlation between these groups, TC, and TIC. Additionally, reports suggest that they exhibit high metabolic versatility, appearing capable of growing on organic matter while also possessing genomic potential for CO₂ fixation by linking nucleoside catabolism to glycolysis–gluconeogenesis (Bulzu et al., 2019). It is then possible that these organisms also utilized this pathway in inorganic carbon rich environments.

In the broader microbial diversity context, syntrophic interactions between Asgard archaea members and other microbes have been described both in environmental studies (Farag et al., 2020; Cai et al., 2021; Zhao and Biddle, 2021) and cultures (Imachi et al., 2020; Rodrigues-Oliveira et al., 2023). Therefore, beyond physical–chemical parameters, the presence of specific syntrophic microbial partners could also influence the Asgard archaeal diversity detected. In this sense, our co-occurrence network analysis (Figure 8) reflect potential relationships among Asgard archaea and different microbes. Indeed, the potential interactions between *Anaerolineae*, *Bathyarchaeia, Desulfobacteria, Dehalococcoidia*, and the Asgard group have been identified before (Hug et al., 2013; Cai et al., 2021). *Desulfobacteria* have also already been described as key partners in the two *Lokiarchaeia* enrichments (Imachi et al., 2020; Rodrigues-Oliveira et al., 2023), supporting the correlation analysis in this study. The distinct major partners of Loki2b (*Nanoarchaeia* and E2) and Loki3 (*Alphaproteobacteria* and UBA1414) indicate diverse physiological activities across different *Lokiarchaeia* subgroups (Yin et al., 2021). Interestingly, *Planctomycetes,* which includes isolates that are capable of aerobic respiration (Vitorino and Lage, 2022), was identified as the *Hodarchaeales*-specific major partner, which is in line with their predicted facultative aerobic lifestyle (Liu et al., 2021, Eme et al., 2023). Overall, the co-occurrence patterns of Asgard archaea and other microbes could provide insights into the symbiotic nature of these organisms, providing clues for future cultivation efforts.

When analyzing the qPCR results to better understand the absolute numbers of the *Lokiarchaeia* in these environments, a notable observation arises (Figure 7). In Hiddensee, there is a lack of vertical stratification in the Asgard archaea community. Conversely, in Lake Techirgiol and Piran, higher absolute numbers are evident near the surface, gradually decreasing with depth. In these latter environments, a decrease in absolute numbers accompanies an increase in relative abundance with depth. This trend reflects a decline in biomass for the overall microbial community as depth increases, coupled with reduced diversity of other organisms. As a consequence, despite the diminishing absolute numbers, the lower depths exhibit higher Asgard archaea relative abundance, an observation consistent with previous findings (Jørgensen et al., 2013; Orsi et al., 2020). Contrastingly, the Hiddensee sediment cores present a different scenario, indicating a lack of stratification. For this sample, we observe consistent absolute and relative abundance values across all depths. It has been suggested that gene expression levels in lokiarchaeal populations appear to be significantly influenced by depth in marine sediments, where genes related to growth, fermentation, and H_2_-dependent carbon fixation show the highest expression under the most reducing conditions (Orsi et al., 2020), and it is possible that such factors influenced the distribution patterns observed here.

## Conclusion

Our analysis revealed pronounced differences in microbial community structure among Hiddensee, Lake Techirghiol, and Piran. These disparities were evident not only at the overall prokaryotic community level but also within the Asgard archaea population. *Lokiarchaeia* and *Thorarchaeia* emerged as predominant groups across all sampled environments, highlighting their widespread distribution. However, the metabolic diversity within these taxonomic ranks suggests adaptations to specific environmental conditions. Our co-occurrence network analysis indicates potential syntrophic relationships between Asgard archaea and other microbes. Finally, we would like to highlight that this study focuses on the Asgard archaea community profile in easy to access shallow sediment environments in a field where most data come from deep marine samples. Additionally, we examined these communities across a depth gradient within the same sediment cores, an approach that is also rare in the existing literature. We also applied an up-to-date taxonomic framework, based on ASV clustering on the latest reference trees, which ensured a more precise classification. Future studies exploring additional environmental parameters, whole genome sequencing, and cultivation efforts will help us gain deeper insights into Asgard archaea ecology.

## Data Availability

The datasets presented in this study can be found in online repositories. The names of the repository/repositories and accession number(s) can be found in the article/Supplementary material.
