# Supplementary material for: Diversity and environmental distribution of Asgard archaea in shallow saline sediments
Source: Front Microbiol. 2025 Mar 18;16:1549128. doi: 10.3389/fmicb.2025.1549128 (PMC11958966; doi:10.3389/fmicb.2025.1549128)
Supplement: Supplementary file 2 [file Image_1.pdf]

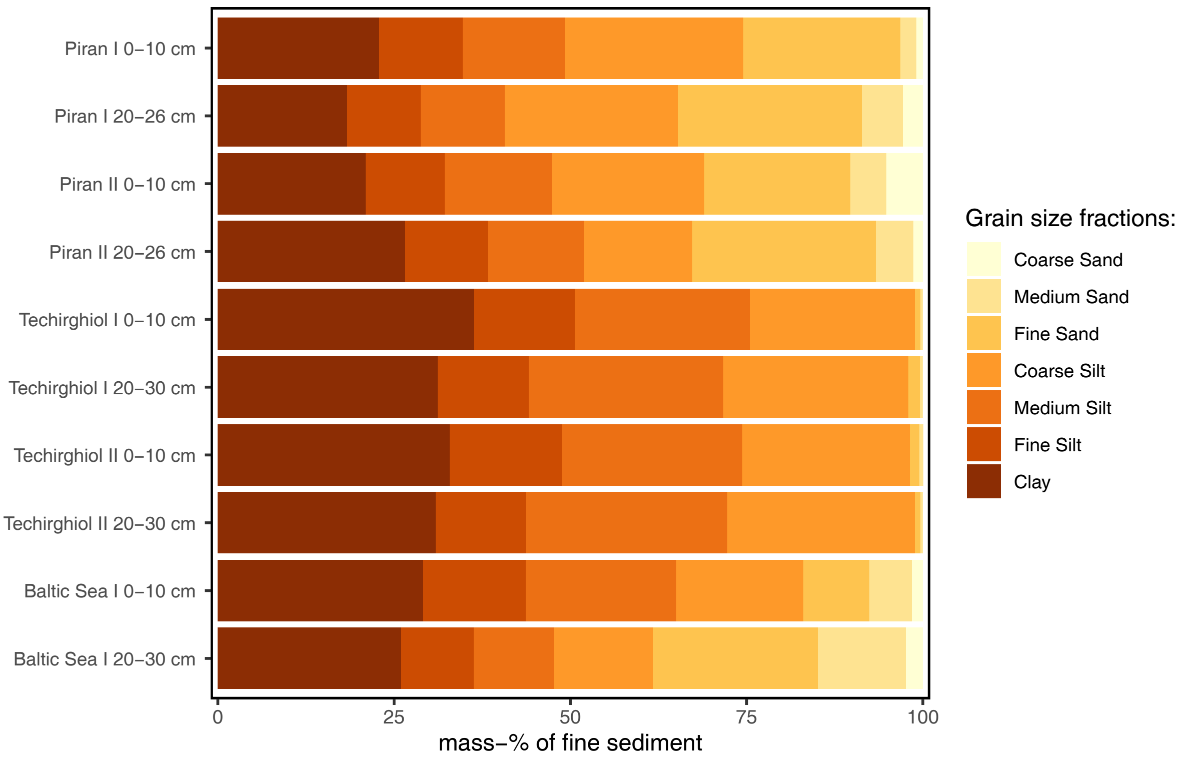

Supplementary Figure 1. Grain Size values measured at different depths in Hiddensee, Piran and Techirgiol
