# Supplementary material for: Diversity and environmental distribution of Asgard archaea in shallow saline sediments
Source: Front Microbiol. 2025 Mar 18;16:1549128. doi: 10.3389/fmicb.2025.1549128 (PMC11958966; doi:10.3389/fmicb.2025.1549128)
Supplement: Supplementary file 3 [file Image_2.pdf]

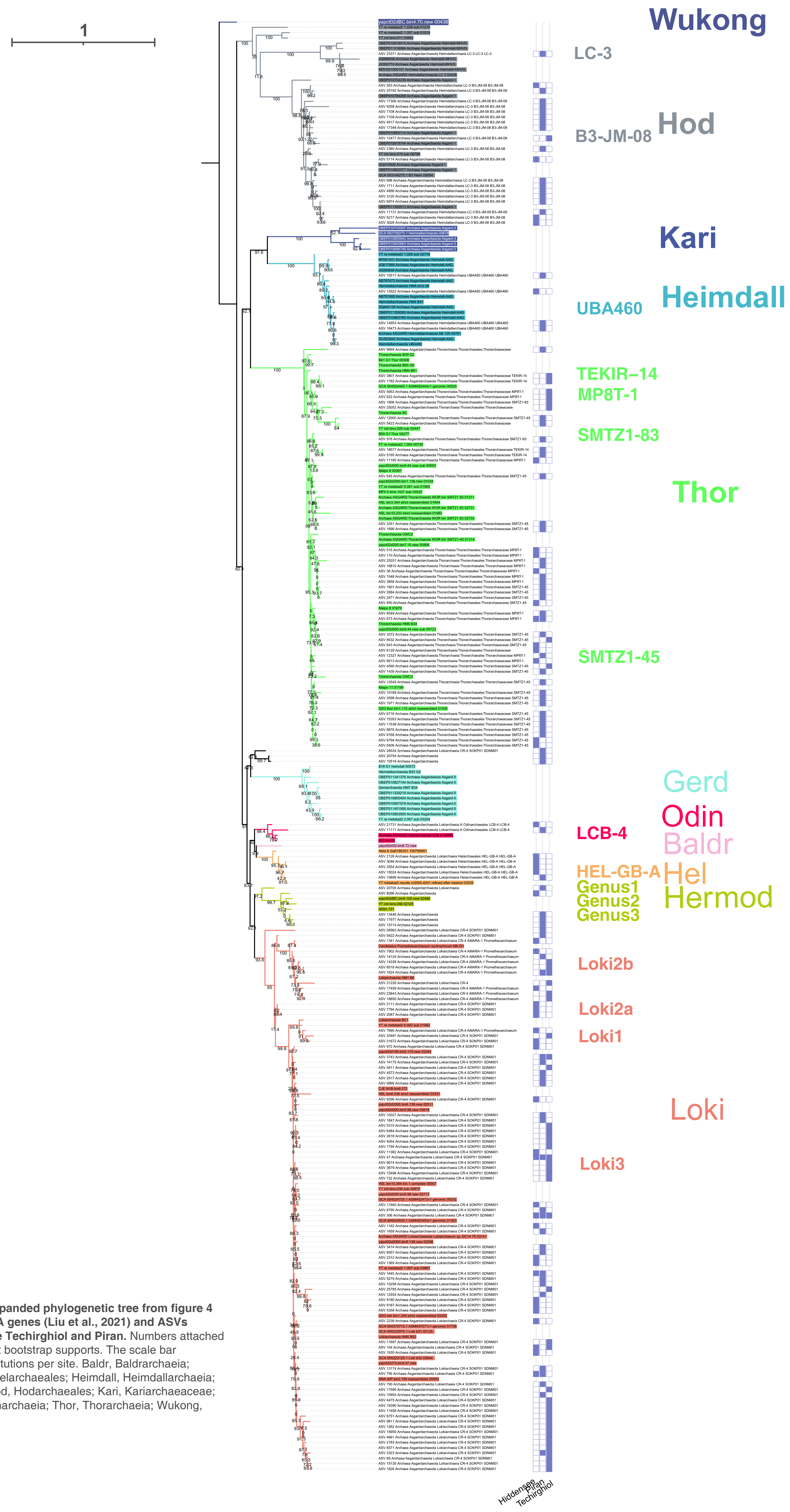

Supplementary Figure 2. Expanded phylogenetic tree from figure 4 of Asgard archaea 16S rRNA genes (Liu et al., 2021) and ASVs detected in Hiddensee, Lake Techirghiol and Piran. Numbers attached to the branches show ultrafast bootstrap supports. The scale bar indicates the number of substitutions per site. Baldr, Baldrarchaeia; Gerd, Gerdarchaeales; Hel, Helarchaeales; Heimdall, Heimdallarchaeia; Hermod, Hermodarchaeia; Hod, Hodarchaeales; Kari, Kariarchaeaceae; Loki, Lokiarchaeia; Odin, Odinarchaeia; Thor, Thorarchaeia; Wukong, Wukongarchaeia.
