# Supplementary material for: Diversity and environmental distribution of Asgard archaea in shallow saline sediments
Source: Front Microbiol. 2025 Mar 18;16:1549128. doi: 10.3389/fmicb.2025.1549128 (PMC11958966; doi:10.3389/fmicb.2025.1549128)
Supplement: Supplementary file 4 [file Image_3.pdf]

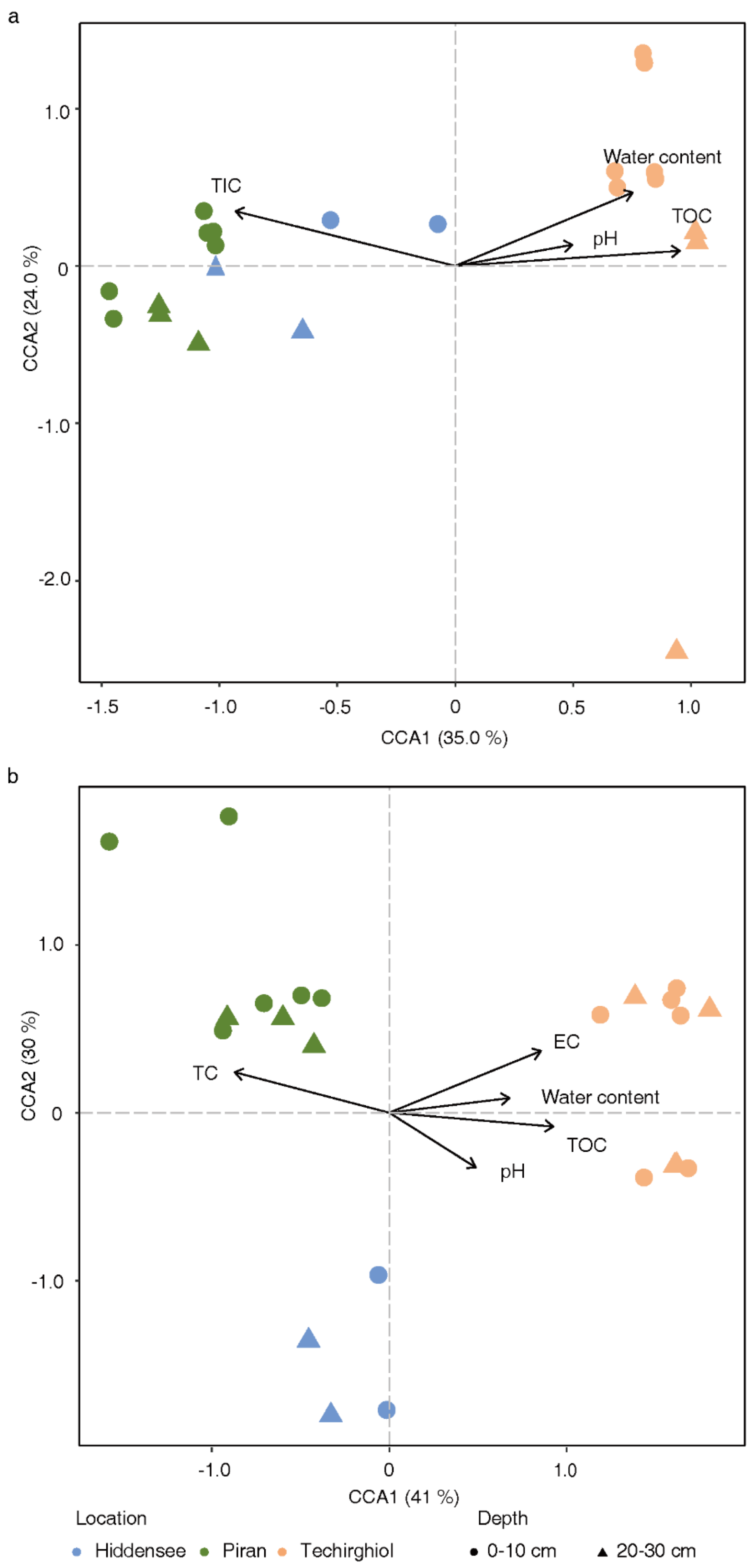


**Supplementary Figure 3. Canonical Correspondence Analysis (CCA) biplots based on the whole microbial community (a) and Asgard archaeal 16S rRNA genes (b).**
